# Antimicrobial resistance pattern of methicillin-resistant *Staphylococcus aureus* isolated from sheep and humans in Veterinary Hospital Maiduguri, Nigeria

**DOI:** 10.14202/vetworld.2022.1141-1148

**Published:** 2022-04-30

**Authors:** Solomon Jauro, Mark M. Hamman, Kefas D. Malgwi, Jasini A. Musa, Yusuf B. Ngoshe, Isa A. Gulani, Iliya D. Kwoji, Ibrahim Iliya, Mustapha B. Abubakar, Folorunso O. Fasina

**Affiliations:** 1 Department of Veterinary Microbiology, Faculty of Veterinary Medicine, University of Maiduguri, Maiduguri, Borno, Nigeria; 2 Veterinary Teaching Hospital, University of Maiduguri, Maiduguri, Borno, Nigeria; 3 Department of Production Animal Studies (Epidemiology Section), Faculty of Veterinary Science, University of Pretoria, South Africa; 4 Department of Veterinary Medicine, Faculty of Veterinary Medicine, University of Maiduguri, Maiduguri, Borno, Nigeria; 5 Department of Pharmaceutical Chemistry, Faculty of Pharmacy, University of Maiduguri, Maiduguri, Borno, Nigeria; 6 Food and Agriculture Organization, Dar es Salaam, Tanzania; 7 Department of Veterinary Tropical Diseases, University of Pretoria, South Africa

**Keywords:** antimicrobial resistance, humans, methicillin-resistant *Staphylococcus aureus*, sheep

## Abstract

**Background and Aim:**

Methicillin-resistant *Staphylococcus aureus* (MRSA), an important opportunistic pathogen, is a Gram-positive coccus known to be resistant to β-lactam antibiotics. Its virulence depends on a large range of factors, mainly extracellular proteins, such as enzymes and exotoxins, that contribute to causing a wide range of diseases in human and animal species. The major reasons for the success of this pathogen are its great variability, which enables it to occur and thrive at different periods and places with diverse clonal types and antibiotic resistance patterns within regions and countries. Infections caused by antibiotic-resistant *S. aureus* bring about serious problems in the general population (humans and animals). Infections with these pathogens can be devastating, particularly for the very young, adults and immunocompromised patients in both humans and animals. This study aimed to determine the presence of MRSA in both apparently healthy and sick sheep brought to the veterinary hospital as well as veterinary staff and students on clinical attachment in the hospital.

**Materials and Methods:**

A total of 200 nasal swab samples were collected aseptically from sheep and humans (100 each) for the isolation of MRSA. The samples were processed by appropriately transporting them to the laboratory, then propagated in nutrient broth at 37°C for 24 h followed by subculturing on mannitol salt agar at 37°C for 24 h, to identify *S. aureus*. This was followed by biochemical tests (catalase and coagulase tests) and Gram staining. MRSA was isolated using Clinical Laboratory Standard Institute (CLSI) guideline and confirmed by plating onto Oxacillin (OX) Resistance Screening Agar Base agar. The antimicrobial susceptibility pattern of the MRSA isolates was determined using the disk diffusion method against 12 commonly used antimicrobial agents.

**Results:**

The total rate of nasal carriage of *S. aureus* and MRSA was found to be 51% and 43% in sheep and humans, respectively. The MRSA prevalence in male and female sheep was 18% and 8%, while 9% and 8% were for male and female human samples, respectively. The antimicrobial susceptibility test showed 100% resistance to OX, cefoxitin, oxytetracycline, cephazolin, and penicillin-G (Pen) by MRSA isolates from humans. Conversely, there was 100% susceptibility to ciprofloxacin, imipenem, and gentamicin; for linezolid (LZD), it was 87.5%, norfloxacin (NOR) (71%), and erythromycin (ERY) (50%) susceptibility was recorded. The MRSA isolates from sheep recorded 100% resistance to the same set of drugs used for human MRSA isolates and were equally 100% susceptible to gentamicin, imipenem, LZD, ciprofloxacin, NOR (92%), and ERY (50%).

**Conclusion:**

This study determined the presence of MRSA in sheep and humans from the Veterinary Hospital, Maiduguri. It appears that certain drugs such as ciprofloxacin, imipenem, and gentamicin will continue to remain effective against MRSA associated with humans and sheep. Reasons for the observed patterns of resistance must be explored to reduce the burdens of MRSA resistance. Furthermore, the present study did not confirm the MRSA resistance genes such as *mecA* and *spa* typing to ascertain the polymorphism in the X-region using appropriate molecular techniques. Hence more studies need to be conducted to elucidate these findings using robust techniques.

## Introduction

*Staphylococcus aureus* is the most abundant skin-colonizing bacteria and the most important cause of nosocomial and community-associated skin infections [1]. It is also an important opportunistic pathogen, and its virulence depends on a large array of factors, mainly extracellular proteins, such as enzymes and exotoxins, that contribute to causing a wide range of diseases in human and animal species [2,3]. The primary reason for the success of this pathogen is its significant variability, which enables it to occur at different periods and places with diverse clonal types and antibiotic resistance patterns within regions and countries [4]. Infections caused by antibiotic-resistant *S. aureus* bring about serious problems in the general population. Such infections can be devastating, particularly for the very young, adults and immunocompromised patients [5]. Some *S. aureus* has undergone genetic modification that has resulted in antibiotic-resistant strains. Over the past 50 years, this organism has become resistant to penicillin due to the production of β-lactamases enzymes that hydrolyze β-lactams antibiotics such as penicillin, thereby rendering them biologically inert. However, in <5 years, resistance to methicillin was developed by *S. aureus* in 1961 [6,7]. Methicillin-resistant *Staphylococcus aureus* (MRSA) possesses reduced affinities for binding to β-lactam antibiotics by producing a specific penicillin-binding protein, PBP2 (or PBP2a), resulting in β-lactam antibiotic resistance [8]. The resistance acquired by Methicillin (oxacillin [OX])-resistant *S. aureus* is extended to most of the commonly used antimicrobial agents, including the aminoglycosides, macrolides, chloramphenicol, tetracycline, and fluoroquinolones [9,10]. They are also reported to be resistant to all cephems, cephalosporins, and other β-lactams (such as amoxicillin-clavulanic acid, ticarcillin-clavulanic acid, ampicillin-sulbactam, carbapenems, and the piperacillin-tazobactam) regardless of the *in vitro* test results obtained with those agents [11].

MRSA has traditionally been considered a hospital-associated methicillin-resistant *Staphylococcus aureus* (HA-MRSA) pathogen [12]. The risk factors for HA-MRSA usually include prolonged hospital stay and antibiotic treatment, surgical intervention, and/or close contact with infected or colonized MRSA-positive individuals [12]. Until the 1990s, MRSA infections were rarely observed in extramural communities [13]. However, in the mid-1990s, MRSA strains were increasingly reported in healthy people without a history of healthcare-associated risk factors [13]; such cases were referred to as community-associated MRSA (CA-MRSA). Research has shown that the genetic makeup of HA-MRSA and CA-MRSA differs significantly [13]. Recently, there have been reports of livestock-linked MRSA isolation in a 6-month-old girl admitted to a hospital for invasive surgery in the Netherlands [14]. Again, the isolates were identified as a new MRSA lineage, different from HA-MRSA, and CA-MRSA with the sequence type 398 grouped within the clonal complex 398; hence, they are called livestock-associated MRSA [15,16].

This study aimed to investigate the presence of MRSA in sheep and humans (veterinary staff and students on clinical attachment), and to determine the antimicrobial resistance patterns of the isolated microorganism.

## Materials and Methods

### Ethical approval and Informed consent

Ethical approval was not required for this study; however, samples were collected as per standard sample collection procedure without unnecessary harm to animals. Verbal consent of the research subjects (Animal owners and Hospital workers and students) was sought before samples were collected.

### Study period and location

The study was conducted from August to December, 2020. This study was achieved by conveniently sampling nasal swabs of human subjects (Hospital workers and those on clinical attachment) and apparently healthy/sick animals brought to the hospital for a routine clinical checkup. The study was conducted at the Veterinary Hospital, Maiduguri, Borno State, Nigeria. Maiduguri is the largest city and capital of Borno State, Nigeria. The hospital is the only major state-owned Veterinary Hospital in the city; it provides veterinary services to all the inhabitants of Maiduguri.

### Sampling

Using a convenient sampling technique, nasal swabs were collected from apparently healthy and sick sheep (n=100) brought for routine examination or treatment and from humans (n=100). The human samples originated from veterinary hospital staff and students on clinical attachment. The swabs were aseptically collected using sterile swab sticks (Medical Wire and Equipment Medical wire, Corsham, Wiltshire, England) from the nasal mucosa. Each swab sample was packaged appropriately and put together in a zipped plastic bag, and then they were immediately transported on an icepack in a cooler box at the temperature of 4°C to the Department of Veterinary Medicine Research Laboratory of the University of Maiduguri for bacteriological assay.

### Bacterial isolation

Each swab stick was immediately inoculated onto mannitol salt agar (Oxoid Limited, England) plates and incubated at 37°C for 24 h. The organisms were isolated aseptically and characterized using established microbiological methods, including colonial morphology, Gram stain characteristics, catalase, and coagulase tests [17]. Isolates that were Gram-positive cocci, catalase-positive, and coagulates human plasma were considered *S. aureus*. The main methods are coagulate and catalase test as mentioned above. Therefore, no need to mention the additional biochemical test because they are not so significant in *S. aureus* identification.

### Detection of MRSA

The MRSA detection was done based on the Clinical Laboratory Standard Institute (CLSI) (Clinical and Institute) guideline. The methicillin resistance of *S. aureus* isolates was confirmed using oxacillin resistance screening agar base (ORSAB) from Oxoid Limited. ORSAB is a selective chromogenic medium for detecting and differentiating MRSA from methicillin-susceptible *S. aureus* (MSSA). The media were prepared, and the selective supplement was added to ORSAB, according to the manufacturer’s instructions. *Staphylococcus* isolates were inoculated on ORSAB and incubated for 24-48 h at 37°C to determine the presence of MRSA.

### Antimicrobial susceptibility test (AST)

The *in vitro* antimicrobial susceptibility pattern of MRSA isolates was carried out using 12 antimicrobial disks, namely, erythromycin (ERY) 15 μg/disk, OX 1 μg/disk, trimethoprim/sulfamethoxazole 2 μg/disk, linezolid (LZD) 30 μg/disk, imipenem 10 μg/disk, cephazolin (KZ) 30 μg/disk, norfloxacin (NOR) 10 μg/disk, ciprofloxacin 5 μg/disk, gentamicin 10 μg/disk, Pen 10 units, cefoxitin (FOR) 10 μg/disk, and oxytetracycline (OT) 30 μg/disk; all the antimicrobial disks used were purchased from Oxoid. The test was performed according to the Kirby–Bauer disk diffusion method as described in CLSI [18] guideline. The standardized overnight culture of each isolate was constituted to McFarland turbidity standard (containing approximately 10^6^ colony-forming units/mL). Then, it was used to flood the surface of Mueller-Hinton agar (Sigma^®^ Saint Louis, MO, USA) plates and the excess was drained off, then allowed to dry while the Petri dish lid was in place. The standard antimicrobial disks were aseptically placed on the inoculated plates and allowed to stand for 1 h. The plates were then incubated at 37°C for 18-24 h. The diameter of the zone of inhibition produced by each antimicrobial disk was measured with a ruler in millimeters. Breakpoints and interpretative criteria for susceptibility/resistance were based on the performance standards for antimicrobial disk susceptibility tests approved by the CLSI [18].

### Statistical analysis

Simple descriptive statistics and one-way analysis of variance were used to analyze the data generated from this study using GraphPad Prism version 6.01 software (GraphPad Software Inc., California, USA). The figures were created using the same version of GraphPad Prism. Values with p*<*0.05 are considered statistically significant.

## Results

### *S. aureus* and MRSA detection

**Figure-1:**
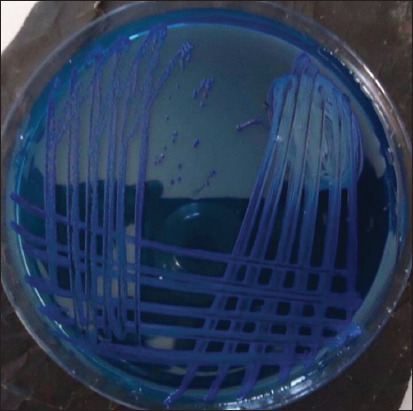
Culture plate showing the deep blue coloration of Methicillin-resistant *Staphylococcus aureus* on oxacillin resistance screening agar base media.

In this study, 200 nasal swabs were collected from sheep and humans and analyzed for nasal carriage of *S. aureus* and MRSA using established techniques. Isolated MRSA confirmation was done using certain characteristic colonial morphology (deep blue coloration) (Figure-1), whereas those that showed no evidence of growth were considered to be MSSA. Out of 200 samples from sheep and humans, 101/200 (51%) were positive for *S. aureus*, with a significant difference (p*<*0.05) observed between the human (40%) and sheep (61%) samples. About 22% (n=43) of the overall 200 samples were positive for MRSA strains. Out of the 100 sheep samples collected, 26 (26%) were positive for MRSA and were from sheep of various age groups, and 17 (17%) of the human samples were positive for MRSA from 100 humans sample collected, as indicated in Table-1. The incidence of *S. aureus* and MRSA among sheep and humans based on sexes is shown in Table-2. There was no significant difference (p>0.05) observed in the sex distribution of the 61 (61%) *S. aureus* positive from the 100 sheep samples, out of which 42 (63%) were male and 19 (58%) from females. Whereas 18 (27%) of the male sample and 8 (24%) of the females were found to be positive for MRSA strains from the overall MRSA positive [26 (26%)] among *S. aureus* from the sheep sample (Table-2). On the other hand, 40 of the humans sampled in this study that was found to be colonized with *S. aureus* showed no significant difference (p*>*0.05*)* among the sexes, with the male having 23 (41%) and females having 17 (39%) while 9 (16%) were positive for MRSA strains among male human samples, and 8 (18%) of MRSA strains were recorded among female human samples. Overall, no significant difference (p>0.05) was observed among human and animal populations in this study regarding sex distribution and predisposition to both *S. aureus* and MRSA. This indicates that both sexes are equally predisposed to the pathogens and have to share the same risk of a carriage. The incidence of nasal carriage of *S. aureus* in apparently healthy and sick sheep differed. Of the 61% of the entire sample that harbors *S. aureus*, 48 (73%) of the sick and 13 (38%) of the apparently healthy sheep were positive for *S. aureus* (p<0.05), 26 (26%) of the entire sheep samples were positive for MRSA strains with a statistical significance (p<0.05) among sheep of different health status, 24 (36%) of the sick sheep and 2 (6%) of the healthy sheep were found to harbor MRSA, as shown in Table-3.

**Table 1:** *S. aureus* and MRSA among sheep and humans.

Breed	*S. aureus*		MRSA	
	
	Number of samples	Number of positive samples (%)	Number of samples	Number of positive samples (%)
Sheep	100	61 (61)^a^	100	26 (26)^a^
Humans	100	40 (40)^a^	100	17 (17)^b^
Total	200	101 (51)	200	43 (22)

Values along the same column with superscript

a are significant, values along the same column with superscript^a^ and

b are not significant, *S. aureus*=*Staphylococcus aureus*, MRSA=Methicillin-resistant *Staphylococcus aureus*

**Table 2:** Sex distribution of *S. aureus* and MRSA among sheep and humans.

Sex	Sheep			Human		
	
	Number of samples	Number of samples positive for *S. aureus* (%)	Number of samples positive for MRSA (%)	Number of Samples	Number of samples positive for *S. aureus* (%)	Number of samples positive for MRSA (%)
Male	67	42 (63)^a^	18 (27)^a^	56	23 (41)^a^	9 (16)^a^
Female	33	19 (58)^b^	8 (24)^b^	44	17 (39)^b^	8 (18)^b^
Total	100	61 (61)	26 (26)	100	40 (40)	17 (17)

Values along the same column with superscript

a and

b are not significant, *S. aureus*=*Staphylococcus aureus*, MRSA=Methicillin-resistant *Staphylococcus aureus*

**Table 3:** *S. aureus* and MRSA in apparently healthy and sick sheep.

Health status	Total number of tested (n=100)	*S. aureus* number of positive (%)	MRSA number of positive (%)
Sick	66	48 (73)^a^	24 (36)^a^
Healthy	34	13 (38)^a^	2 (6)^a^
Total	100	61 (61)	26 (26)

Values along the same row with superscript

a are significant, *S. aureus*=*Staphylococcus aureus*, MRSA=Methicillin-resistant *Staphylococcus aureus*

### AST

**Figure-2:**
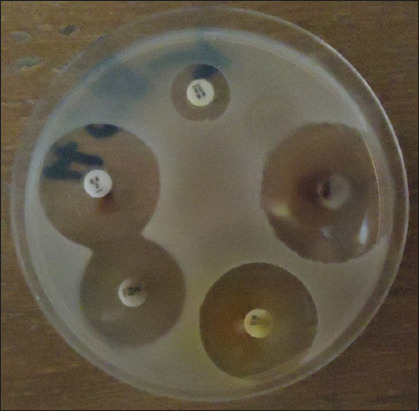
Culture plate showing the zone of inhibition by different antimicrobial agents against Methicillin-resistant *Staphylococcus aureus* on Mueller-Hinton agar.

**Figure-3:**
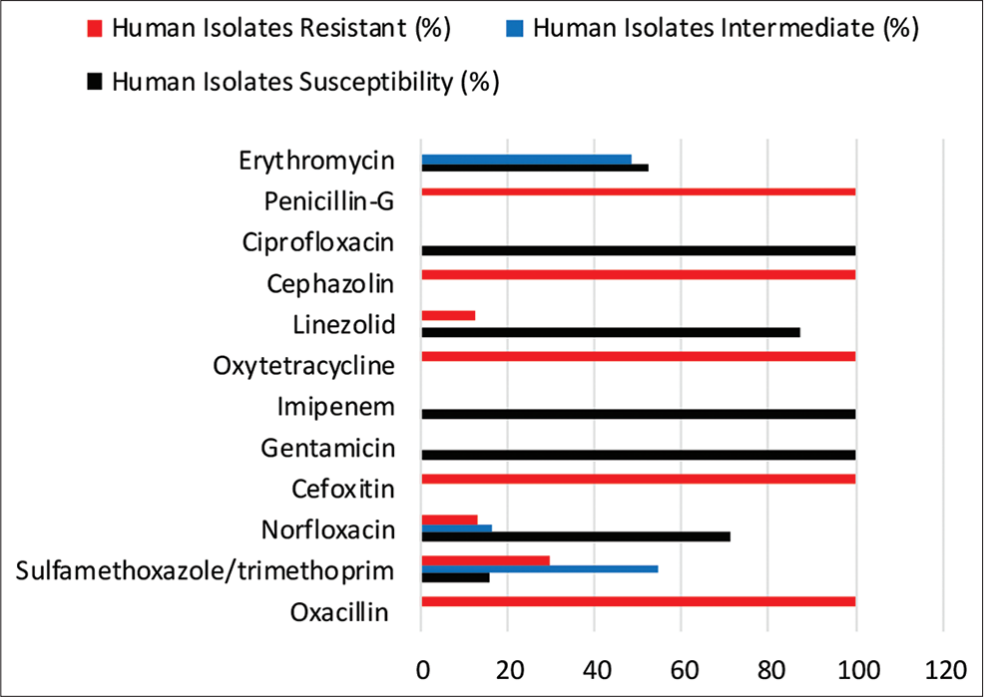
Antimicrobial susceptibility patterns of Methicillin-resistant *Staphylococcus aureus* isolates from humans in Maiduguri.

**Figure-4:**
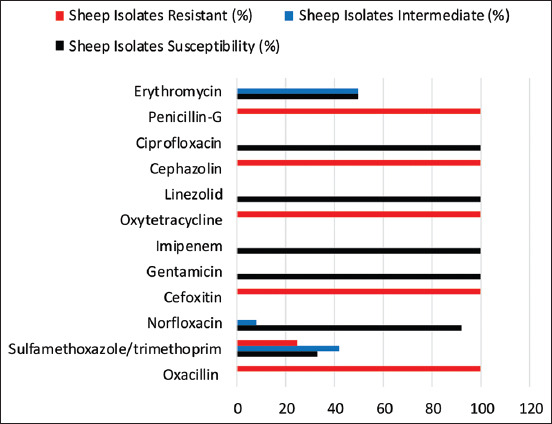
Antimicrobial susceptibility patterns of Methicillin-resistant *Staphylococcus aureus* isolates from sheep in Maiduguri.

The AST of the MRSA isolated from humans and animals was determined by measuring the diameter of the zone of inhibition, as shown in Figure-2. The AST of the MRSA isolated from humans is represented in Figure-3, the isolates showed the maximum level of susceptibility to gentamicin (100%), imipenem (100%), and ciprofloxacin (100%), followed by LZD (87.5%) and NOR (71%), but to ERY, they showed 52.5% susceptibility and 16% against sulfamethoxazole/trimethoprim (SXT). The isolates recorded the maximum level of resistance of 100% to OX, FOR, OT, KZ, and Pen. The AST of the strains of MRSA isolated from sheep is shown in Figure-4. The isolates revealed 100% susceptible to gentamicin, imipenem, LZD, and ciprofloxacin, followed by NOR (92%), ERY (50%), and SXT (42%). The sheep isolates showed a high level of resistance to multiple antimicrobial agents, which include; OX (100%), FOR (100%), OT (100%), KZ (100%), Pen (100%), and SXT (25%). The multidrug-resistant profile of MRSA isolates for sheep and humans is represented in Table-4. It was observed in this study that MRSA isolates from both sheep and humans are multidrug-resistant MRSA. The majority of the MRSA isolates from humans and sheep have shown resistance to ≥3 antibiotics, and only four isolates, two each from the sheep, showed resistance to eight different antibiotics.

**Table 4:** Multidrug resistance profile of MRSA isolated from sheep and humans.

Antibiotics	Sheep isolates Number (%)	Human isolates Number (%)
Pen, OX, and OT	26	20
Pen, OX, OT, and FOR	26	17
Pen, OX, OT, KZ, and FOR	20	20
Pen, OX, OT, FOR, KZ, and LZP	7	11
Pen, OX, OT, FOR, KZ, SXT, and NOR	0	2
Pen, OX, E, OT, NOR LZP, KZ, and FOR	0	2

Pen=Penicillin-G, OX=Oxacillin, ERY=Erythromycin, OT=Oxytetracycline, NOR=Norfloxacin, LZD=Linezolid, KZ=Cephazolin, FOR=Cefoxitin, SXT=Sulfamethoxazole/trimethoprim, MRSA=Methicillin-resistant *Staphylococcus aureus*

## Discussion

The emergence of antimicrobial resistance in Gram-positive bacteria has been a source of concern worldwide, and options for the treatment of infections and pathogens with antimicrobial agents are becoming fewer and scarcer. This study primarily investigated the nasal carriage of MRSA among sheep brought to the veterinary teaching hospital and among veterinary staff and students attending to the animals. We obtained a nasal carriage rate of 17% for MRSA in humans and 26% in sheep. Sergelidis *et al*. [19] reported 22.2.% MRSA among livestock in Greece, which is slightly different from the finding of this study. Okon *et al*. [20] reported 29% MRSA strain isolated among ruminants slaughtered for consumption in Maiduguri abattoir and ewes his findings closely at par with the 26% MRSA reported in this study. The presence of MRSA obtained among the veterinary staff and the students as well as among the sheep possesses great dissemination of MRSA in the community due to the environmental setting of rearing a small number of animals in the household very to humans.

The MRSA nasal colonization rate among veterinary staff and students on clinical attachment was 17% which is in line with the findings of Ansari *et al*. [21] reported a 15% MRSA nasal colonization rate among preclinical students in Nepal. In addition, another study also reported 18% among veterinary staff in a small animal referral hospital in the UK [22]. However, our findings contradict and exceed 7% reported among veterinary staff by Espadale *et al*. [23], 6.5% reported among veterinary personnel [20], 3.4% reported among contact people handling animals in households, most of which work in the veterinary profession [24], and 1.1% was also reported among sheep farmers in Southern Italy [25]. Similarly, it exceeded human health care workers, where 6.2% was found in France and 6% in Turkey [26,27]. The differences in the MRSA colonization or infection rate among animals and humans may likely be due to the difference in contact hours between humans and animals, the timing and site of sample collections, previous antibiotic therapy, geographical location, and the protocols applied to search for carriage (sites tested and enrichment protocols such as culture method) and antibiotic usage restriction law in the study area. Furthermore, the observation in the present study could probably suggest the possible occurrence of community-acquired MRSA and livestock-associated MRSA in the study area due to the lack of strict laws guiding against the unnecessary use of antibiotics in the study area. Based on the findings in this study, further study is currently ongoing to establish more details and classify the MRSA circulating in the study.

The MRSA isolates in this study showed a varying level of susceptibility and resistance to different antibacterial agents used for the AST. The highest level of resistance (100%) was observed in OX, FOR, OT, KZ, and Pen. The 100% resistance to Pen is in line with the reports of Lee [28], Mai-Siyama *et al*. [20], and Suleiman *et al*. [29], who reported 100%, 96.1%, and 90.2%, respectively, in their findings. Suleiman *et al*. [29] also reported 100% resistance to methicillin which agrees with the finding in this study of 100% resistance shown by the isolates to OX, a member of the same family with methicillin. The report of 100% resistance by the isolates to OT in this study slightly agrees with the reports by Al-Mohana *et al*. [30], 90.2%, Suleiman *et al*. [29], 85%, and Mai-Siyama *et al*. [20], 81.8%. The high resistance may be due to the acquisition and expression of the *mecA* gene or the thickening of the isolates’ cell walls [31]. The excessive use of OT in animal husbandry and lack of firm antibiotic use regulation, especially in this study area, might cause the high resistance recorded against OT. Furthermore, antibacterial agents used in animals for therapeutics, food production, and disease prevention may have also facilitated the occurrence of antibiotic resistance in humans [32]. These may explain the varying and multiple resistances observed in this study.

The high susceptibility of 100% observed in imipenem and ciprofloxacin is similar to the earlier findings by Nworie *et al*. [33] and Mohammed [34]. However, Lee [28] reported lower susceptibility of 68.4% of the isolates to imipenem. However, it is worthy of note that imipenem and ciprofloxacin are not commonly used in treating farm animals in the study area. As a result, they both showed the highest level of efficacy on the isolates. This further reiterates the significance of firm regulation on the use of antibiotics. Nworie *et al*. [33] and Mai-Siyama *et al*. [20] reported low/moderate susceptibility to ciprofloxacin (35% and 42.4%) which disagree with the report of this study. However, Mohammed [34] and Udeani *et al*. [35] reported 86% and 88.2% susceptibility to ciprofloxacin, which is slightly lower than the report of this study. However, the result obtained in this study contradicts the reports by Gupta *et al*. [36], who reported ciprofloxacin to be ineffective (with a resistance rate of 99%) against MRSA and Tenguriaa *et al*. [37] also reported 83% resistance to ciprofloxacin in a study conducted in India. The finding of 96% susceptibility to gentamicin recorded in this study is supported by Udeani *et al*. [37] findings which also reported 96% but varied slightly with reports of Ghamba *et al*. [38], Al-Mohana *et al*. [30], and Nworie *et al*. [33], with 90.5%, 73%, and 85%, respectively; these findings are contradicting to 62.5% resistance of MRSA to gentamicin reported by Olowe *et al*. [39]. In this study, the MRSA isolates have shown 71% susceptibility to NOR, which is slightly higher than the 60% reported by Egyir *et al*. [40] but lower than Lee [28] and Udegbunam *et al*. [41], who reported 89.4% and 100%, respectively. There is a high MRSA susceptibility to (aminoglycoside) gentamicin and the fluoroquinolones (NOR) tested in this study. This finding may be due to the absence of resistance-conferring genes in these MRSA strains to these antimicrobial as reported by Polyzou *et al*. [42]. The observed high MRSA susceptibility to aminoglycoside (gentamicin) and fluoroquinolones (NOR) in this study supports some previous reports [43,44]. In addition, the high rate of susceptibility may not be unconnected to the fact that fluoroquinolones are not commonly used in treating food animals, except for poultry and their usage in humans [41]. Thus, the susceptibility of MRSA to non-β-lactam antibiotics may provide a window opportunity for the recommendation of these drugs for the empirical treatment of MRSA strains, thereby reducing sole reliance on the usage of β-lactam antibiotics for the treatment of *mecA* encoding bacteria. LZD has shown 87.5% effectiveness on MRSA in this study. This is slightly similar to the findings of Nworie *et al*. [33] and Egyir *et al*. [40]; they reported 100% susceptibility of their isolates to LZD. The higher level of MRSA susceptibility to LZD tested in this study and other studies may also be due to the absence of resistance-conferring genes of MRSA, or the antimicrobial agent is newly introduced for therapeutic use against this pathogen. Based on our findings, antimicrobial agents such as imipenem, ciprofloxacin, and gentamicin may continue to be effective against MRSA strain isolates from both humans and animals in the study area.

The multidrug resistance pattern in this study has shown that virtually all the MRSA isolates in this study are multidrug-resistant. This assertion is based on Neyra *et al*. [45] study, where they stated that “Multidrug-resistant bacteria are bacterial isolates that are resistant to three or more classes of antimicrobial agents.” The nasal carriage of MRSA by sheep could serve as a reservoir of MRSA, which may pose a serious threat to public health. The MRSA isolates have shown a high resistance rate to some antibacterial agents commonly used in the study area, including OX, FOR, OT, KZ, and Pen.

## Conclusion

It can be concluded that MRSA is present in sheep and humans (veterinary staff) in the Maiduguri and its environment. These present a significant risk in the spread of multidrug-resistant organisms among animals and humans. Furthermore, the current study did not use molecular approaches to confirm MRSA resistance genes such as *mecA* and *spa* type to determine the polymorphism in the X-region. As a result, more research is needed to elucidate these findings using molecular methods.

## Recommendations

There is a need for further study to elucidate the genetic lineages of MRSA circulating among animals and humans, and the capacity of these strains to produce virulence factors, and the risk of animal ↔ human phase bacterial transference is recommended. Furthermore, regular monitoring of MRSA in animals and veterinary staff should be promoted by setting up a comprehensive veterinary surveillance program on antimicrobial resistance. It is further suggested that strict antibiotic prescription policies and laws should be implemented by appropriate health and veterinary authorities to contain the abuse of antibiotics and reduce the acquisition of resistance by pathogens in the study area. Such policies and laws are already being developed consistent with the Nigerian National Action Plan for Antimicrobial Resistance (NAP), 2017-2022 (FMOAE&H, 2017). Finally, the implementation of the activities in the NAP should promote awareness, education, and stewardship of AMR among the populace.

## Authors’ Contributions

SJ and MBA: Designed the study, participated in the technical work, and wrote the manuscript. KDM, YBN, and MMH: Reviewed and edited the manuscript. IAG, JAM, and IDK: Conducted the technical work. MBA and II: Supervised the study. All authors read and approved the final manuscript.
